# Changes in Indices of Steatosis and Fibrosis in Liver Grafts of Living Donors After Weight Reduction

**DOI:** 10.3389/fsurg.2022.827526

**Published:** 2022-05-03

**Authors:** Jaehyuk Choi, YoungRok Choi, Su young Hong, Sanggyun Suh, Kwangpyo Hong, Eui Soo Han, Jeong-Moo Lee, Suk Kyun Hong, Nam-Joon Yi, Kwang-Woong Lee, Kyung-Suk Suh

**Affiliations:** Department of Surgery, Seoul National University College of Medicine, Seoul National University Hospital, Seoul, South Korea

**Keywords:** hepatic steatosis, hepatic fibrosis, fatty liver, living donor, NAFLD, weight loss

## Abstract

**Background:**

A short-term weight reduction program for potential living donors was introduced to reduce the extent of hepatic steatosis prior to liver transplantation. We aimed to investigate changes in non-invasive hepatic steatosis and fibrosis indices among those who completed the program.

**Methods:**

Among 1,950 potential living liver donors between January 2011 and May 2019, 160 living donors joined the weight reduction program. The prospectively collected clinical data of these potential liver donors were analyzed retrospectively. Hepatic steatosis and fibrosis scores were determined using the fatty liver index (FLI), hepatic steatosis index (HSI), and NAFLD fibrosis score (NFS) and compared to MR spectroscopy (MRS) fat fraction results before and after weight reduction.

**Results:**

Thirty-nine potential living donors who had undergone MRS both before and after weight reduction were included in the analysis. Their body weight decreased from 78.02 ± 10.89 kg to 72.36 ± 10.38 kg over a mean of 71.74 ± 58.11 days. FLI, HSI, and MRS values decreased significantly from 41.52 ± 19.05 to 24.53 ± 15.93, 39.64 ± 3.74 to 35.06 ± 3.82, and 12.20 ± 4.05 to 6.24 ± 3.36, respectively. No significant decreases in NFS were observed. There was a significant correlation between the extent of HSI change and the extent of MRS change (R^2^ value = 0.69, *P* < 0.001), although there was no correlation between MRS and FLI.

**Conclusion:**

The weight reduction program significantly improved non-invasive indices of hepatic steatosis over a short period. HSI may allow for prediction of simple decreases in hepatic steatosis.

## Introduction

The prevalence of non-alcoholic fatty liver disease (NAFLD) in the general population ranges from 20 to 30% in Europe (1, 2), and is as high as 46% in the United States (3). In Korea, the prevalence of NAFLD among potential donors is also increasing due to increasing adoption of a sedentary and Westernized lifestyle (4–6). However, NAFLD—which is related to metabolic syndrome—has an adverse effect on liver transplantation. Indeed, NAFLD has been associated with postoperative morbidity and mortality in patients with liver grafts (7, 8).

Although NAFLD in the donor is an exclusion criterion for living donor liver transplantation (LDLT) (9), donors with NAFLD are considered marginal donors because the supply of deceased donors has not met the demand for liver transplantation in Korea. Previous studies have indicated that weight reduction and lifestyle modification can reduce hepatic steatosis in such marginal donors (10–14).

Education on weight reduction and lifestyle modification is being provided to potential living donors with NAFLD to improve outcomes. Potential donors who have successfully reduced weight prior to liver transplantation exhibit comparable outcomes to donors from whom liver transplantation is completed using liver grafts with no NAFLD (11, 15, 16).

Hepatic steatosis can be assessed either *via* liver biopsy or MR spectroscopy (MRS). However, liver biopsy can cause procedure-related complications, and MRS is expensive. Therefore, there is a need for a simple index that can predict the degree of tissue change following weight reduction.

In this retrospective study, we investigated whether indices of hepatic steatosis and fibrosis, which are already in use and can be calculated simply from the donor's data, can predict the extent of NAFLD after weight loss.

## Materials and Methods

### Study Design

This study involved a single-center retrospective analysis of the electronic medical records of 1,950 potential donors between January 2011 and May 2019. Of the 1,950 potential donors, 160 potential donors with hepatic steatosis received recommendations for weight reduction and lifestyle modification programs. Ninety-three of 160 potential donors lost body weight and underwent LDLT. In this study, the fat fraction of MRS was used as the reference value for the extent of hepatic steatosis (17–19). MRS acquired as previous described (20).

The study protocol conformed to the ethical guidelines of the 1975 Declaration of Helsinki as reflected in a prior approval by the appropriate Institutional Review Committee (2011-121-1173) at Seoul National University Hospital.

### Variable Definitions and Data Collection

Baseline body weight prior to weight loss and final weight after weight loss were measured during the first and last MRI sessions, respectively. Alanine transaminase (ALT), aspartate transaminase (AST), gamma-glutamyl-transpeptidase (GGT), platelet count, albumin, waist circumference, weight loss duration, fat fraction on MRS, and body weight were compared between the two measurement sessions.

### Indices

We noninvasively reviewed indices for NAFLD assessment, including six indices [fatty liver index (FLI), hepatic steatosis index (HSI), SteatoTest, lipid accumulation product (LAP), Index of Nash (ION), NAFLD-Liver Fat Score (LFS)] for liver steatosis and eight indices [AST to platelet ratio index (APRI), Fibrosis-4 (FIB-4), FibroTest, Fibrometer NAFLD, Enhanced Liver Fibrosis (ELF), Hepacore, BARD score, NAFLD fibrosis score (NFS)] for liver fibrosis (21, 22). The FLI, HIS, and NFS were selected for this study because these indices can be calculated with only simple laboratory findings, body weight, and circumference using retrospective data.

The FLI is used to predict the degree of hepatic steatosis (23). Scores are derived based on body mass index (BMI), GGT, triglycerides (TG), and waist circumference, as follows: FLI = logistic (0.953 × ln (TG) + 0.139 × BMI + 0.718 × ln (GGT) + 0.053 × waist circumference – 15.745) ×100, where logistic(x) = 1/(1 + e^−*x*^) and ln denotes the natural logarithm. Its lower cutoff is 30, while its upper cutoff is 60 (24, 25).

The HSI predicts the degree of hepatic steatosis and is derived using BMI, diabetes mellitus (DM) status, and AST/ALT ratio, as follows: HSI = 8 × ALT/AST + BMI (+ 2, if DM) (+ 2, if female). Its lower cutoff is 30, while its upper cutoff is 36. A prior ultrasonography study revealed that HIS is correlated with the extent of hepatic steatosis (26, 27).

The NFS is used to predict the degree of hepatic fibrosis and is derived based on age, sex, DM status, platelet count, AST/ALT ratio, and albumin levels, as follows. NFS = −1.675 + 0.037 × age + 0.094 × BMI + 1.13 × DM + 0.99 × AST/ALT ratio – 0.013 × platelet (10^9^) – 0.66 × ALB (g/dl). Its lower cutoff is −1.445, while its upper cutoff is 0.676 (28–30).

### Statistical Analysis

Statistical analyses were performed using SPSS 26.0 (IBM Corp., Armonk, NY, USA). Continuous variables such as body weight, BMI, AST, ALT, GGT, platelet count, albumin, waist circumference, and indices (NFS, FLI, HSI, MRS) before and after weight loss were compared using Student's *t*-tests. Variable distributions were subjected to a normality test prior to the Student's *t*-test. All *p*-values were one-sided, and *P*-values <0.05 were considered statistically significant. Quantitative variables are expressed as the mean ± standard deviation. Linear regression analyses were performed to investigate correlations between each index and the fat fraction obtained via MRS. R^2^ > 0.6 with a *p*-value <0.05 was considered statistically significant. Arrow graphs were drawn using the R ggplot 2 package.

## Results

### Study Population

**Figure 1:**
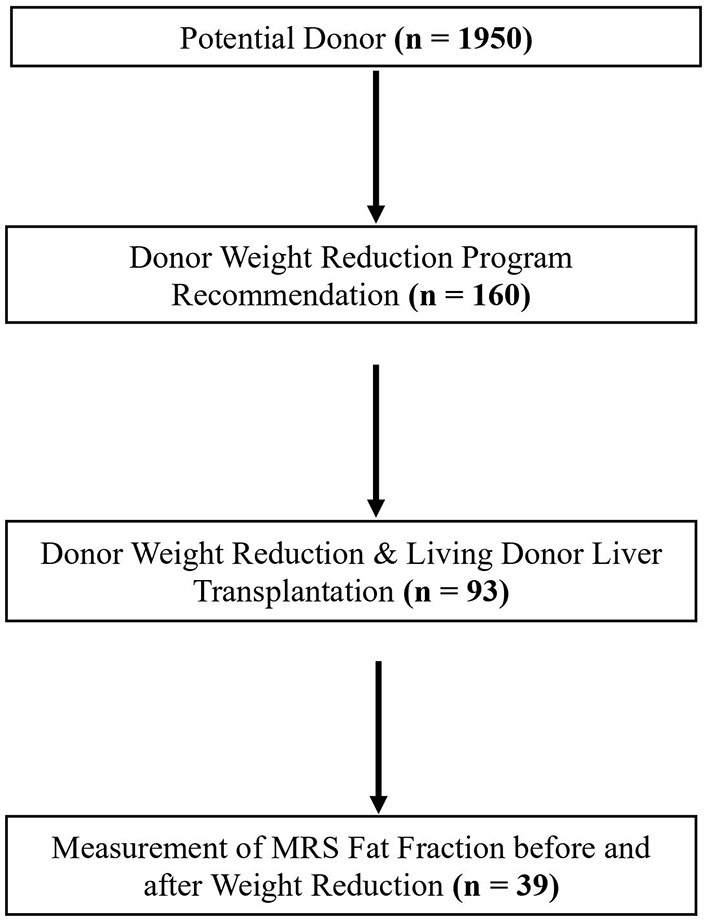
Flow diagram of participant selection.

We analyzed data for 39 of 93 living donors who had undergone assessments of fat fraction before and after weight loss via MRS (Figure 1).

### Patient Characteristics

The mean patient age was 35.03 ± 10.45 years (range: 20–59 years), and 29 patients were male (29/39, 74.4%). No patients had DM, although two donors (5.1%) had hypertension. The mean duration of weight reduction was 71.74 ± 58.11 days (range: 11–298 days). Six donors (15.4%) had a body weight reduction period of over 100 days (Table 1).

**Table 1:** Baseline characteristics of the 39 living donors.

**Variables**	**Before**	**After**	***P*-value**
Sex (male), *n* (%)	29 (74.4%)	-	NA
Age (years)	35.03 ± 10.45	-	NA
Weight (kg)	78.02 ± 10.89	72.36 ± 10.38	<0.001
Height (cm)	169.53	-	NA
BMI (kg/m^2^)	27.10 ± 2.83	25.11 ± 2.60	<0.001
Waist circumference (mm)	909.35 ± 59.58	879.92 ± 61.16	<0.001
DM	0	-	NA
HT, *n* (%)	2 (5.1%)	-	NA
Weight loss duration (days)	71.74 ± 58.11	-	NA

*BMI, body mass index; DM, diabetes mellitus; HT, hypertension*.

Participation in the program was associated with a significant decrease in weight from 78.02 ± 10.89 kg to 72.36 ± 10.38 kg (*P* < 0.001), as well as a significant decrease in waist circumference from 909.35 ± 59.58 mm to 879.92 ± 61.16 mm (*P* < 0.001). Moreover, BMI decreased from 27.10 ± 2.83 kg/m^2^ to 25.11 ± 2.60 kg/m^2^ (*P* < 0.001), and the distribution of BMI changed. Thirty living donors (76.9%) had obesity (BMI > 25 kg/m^2^), eight donors (20.5%) were overweight (25 kg/m^2^ > BMI > 23 kg/m^2^), and one donor' s BMI was 22.89 kg/m^2^, which is nearly borderline. After completing the weight reduction program, 19 donors (48.8%) had obesity (BMI > 25 kg/m^2^), 10 donors (25.6%) were overweight (25 kg/m^2^ > BMI > 23 kg/m^2^), and 10 donors (25.6%) had normal weight (Table 1).

There was no significant difference in AST levels between the baseline and final assessments (21.28 ± 5.88 U/L vs. 19.23 ± 6.58 U/L, *P*-value = 0.167); however, there were significant decreases in ALT (33.08 ± 15.80 U/L vs. 23.10 ± 11.35 U/L, *P* = 0.007) and GGT levels (33.13 ± 18.41 U/L vs. 21.69 ± 10.79 U/L, *P* < 0.001) between the two time points. The number of patients with ALT levels above the normal range (0–40 U/L) changed from 9/39 to 3/39, while the number of patients with GGT levels above the normal range changed from 19/39 to 6/39. There was a significant decrease in albumin levels between the baseline and final assessments (4.52 ± 0.25 g/dl vs. 4.41 ± 0.34 g/dl, *P* = 0.004). However, platelet count did not significantly differ between the two time points (*P* = 1.00) (Table 2).

**Table 2:** Laboratory findings.

**Variables**	**Before**	**After**	***P*-value**
AST (U/L)	21.28 ± 5.88	19.23 ± 6.58	0.167
ALT (U/L)	33.08 ± 15.80	23.10 ± 11.35	0.007
GGT (U/L)	33.13 ± 18.41	21.69 ± 10.79	<0.001
Platelet (×10^9^/L)	256.36 ± 57.66	249.44 ± 58.28	1
Albumin (g/dL)	4.52 ± 0.25	4.41 ± 0.34	0.004

*AST, aspartate aminotransferase; ALT, alanine aminotransferase; GGT, gamma-glutamyl transferase*.

### Index Analysis

There was a significant decrease in HSI between the baseline and final assessments (39.64 ± 3.74 vs. 35.06 ± 3.82, *P* < 0.001). Before weight loss, there were 37 donors (94.9%) in the high HSI group (HSI > 36) and two donors (5.1%) in the intermediate HSI group (36 > HSI > 30). After weight loss, there were 16 donors (41.0%) in the high HSI group (HSI > 36), 21 donors (53.8%) in the intermediate HSI group (36 > HSI > 30), and two donors (5.2%) in the low HSI group (HSI <30) (Table 3).

**Table 3:** Hepatic steatosis & fibrosis indices.

**Variables**	**Before**	**After**	***P*-value**
NFS	−3.45 ± 0.89	−3.22 ± 0.94	1
FLI	41.52 ± 19.05	24.53 ± 15.93	<0.001
HIS	39.64 ± 3.74	35.06 ± 3.82	<0.001
MRS	12.20 ± 4.05	6.24 ± 3.36	<0.001

*NFS, NAFLD fibrosis score; FLI, fatty liver index; HSI, hepatic steatosis index; MRS, MR spectroscopy liver fat fraction; NAFLD, non-alcoholic fatty liver disease*.

There was a significant decrease in FLI between the baseline and final assessments (41.52 ± 19.05 vs. 24.53 ± 15.93, *P* < 0.001). Before weight loss, there were seven donors (17.9%) in the high FLI group (FLI > 60), 27 donors (69.2%) in the intermediate FLI group (60 > FLI > 20), and five donors (12.9%) in the low FLI group (FLI <20). After weight loss, there was one donor (2.6%) in the high FLI group (FLI > 60), while there were 17 donors (43.6%) in the intermediate FLI group (60 > FLI > 20) and 21 donors (53.8%) in the low FLI group (FLI <20).

No significant changes in NFS were observed (*P* = 1).

Measurements of fat fraction obtained via MRS significantly decreased from 12.20 ± 4.05% to 6.24 ± 3.36% (*P* < 0.001) between the two periods. Before weight loss, the fat fraction was 10% or higher in 26 donors, and the maximum value was 22.73%. After weight loss, only four donors had fat fractions of 10% or higher (Table 3).

### Correlation in the Value of Delta Between HSI and MRS

**Figure 2:**
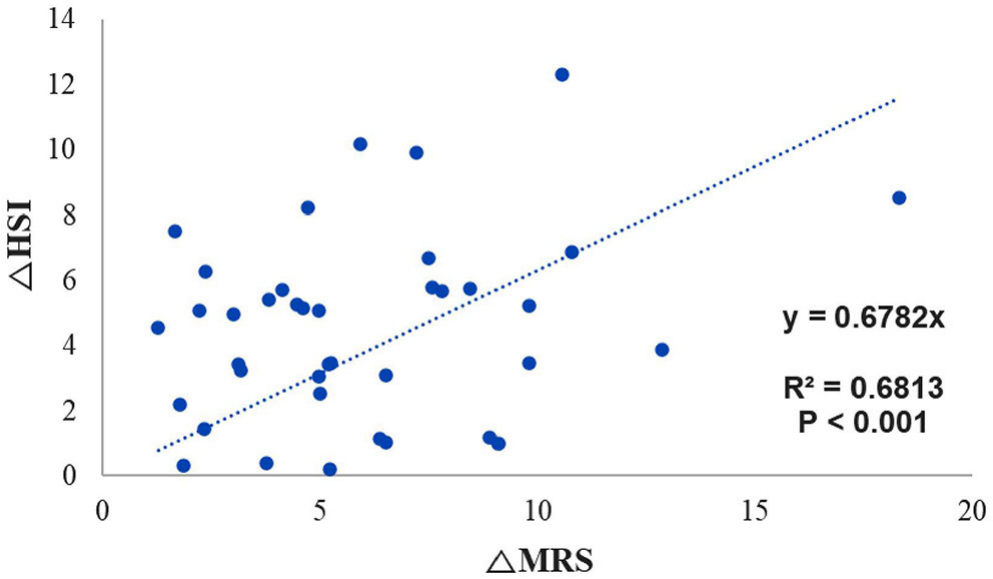
Trend line for identifying the correlation between the change in MRS fat fraction and the change in HSI. MRS, magnetic resonance spectroscopy; HSI, hepatic stenosis index.

There were no significant correlations between the extent of change on MRS and indices, except HSI (R^2^ = 0.69 and *P* < 0.001) (Figure 2).

**Figure 3:**
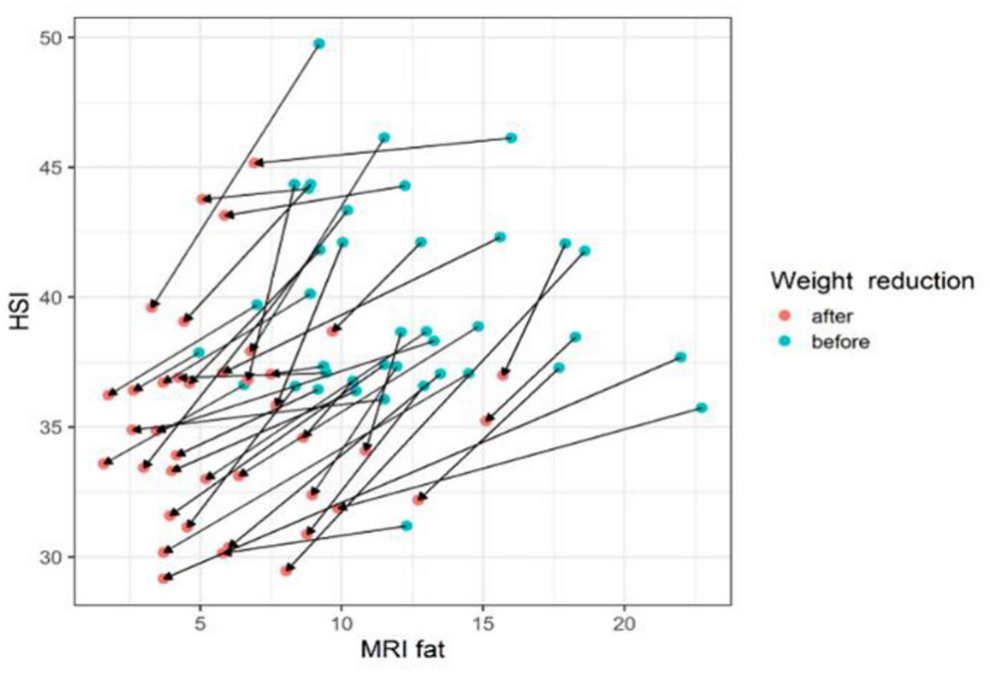
The changes of HIS according to MRI fat before and after body weight reduction. The values of HSI and MRS tend to decrease with weight loss. The line slope representing the change is the ratio of the MRS decrease and the HSI decrease. Therefore, the HSI value changes appropriately according to the change amount of the MRI fat fraction. MRS, magnetic resonance spectroscopy; HSI, hepatic stenosis index. Blue circle, before weight reduction; red circle and arrows, after weight reduction.

**Figure 4:**
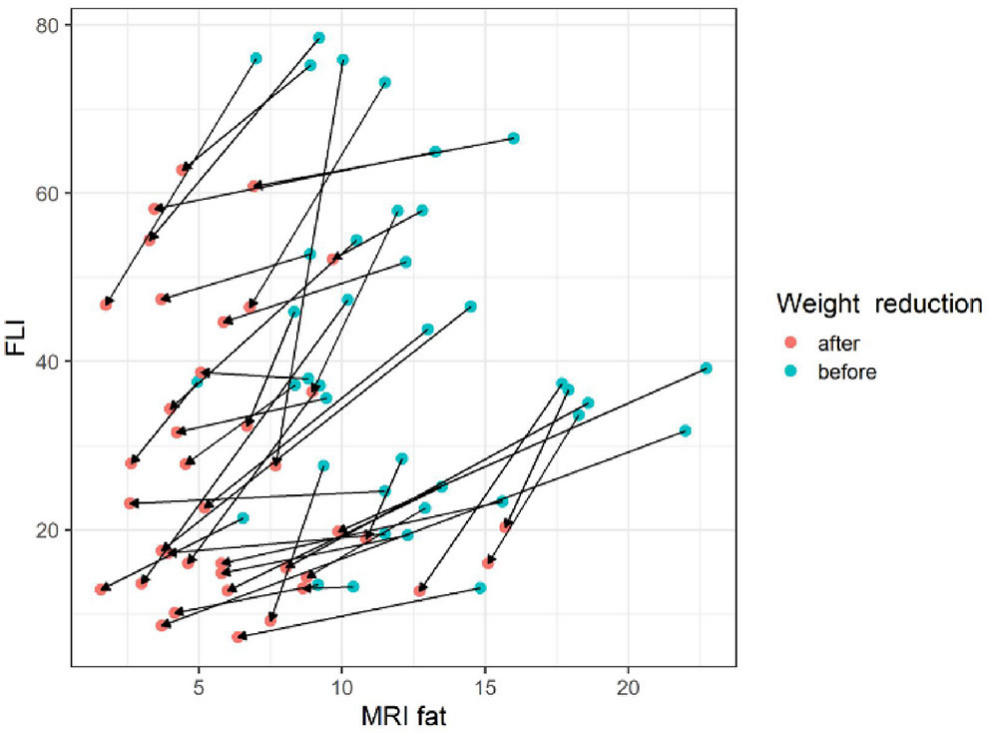
The changes of FLI according to MRI fat before and after body weight reduction. The slope of each line appears in various ways without a particular pattern. Since the amount of change in the FLI value according to the amount of change in the MRS fat fraction is not constant, it appears that the FLI cannot consistently reflect the MRS fat fraction. MRS, magnetic resonance spectroscopy; FLI, fatty liver index. Blue circle, before weight reduction; red circle and arrows, after weight reduction.

**Figure 5:**
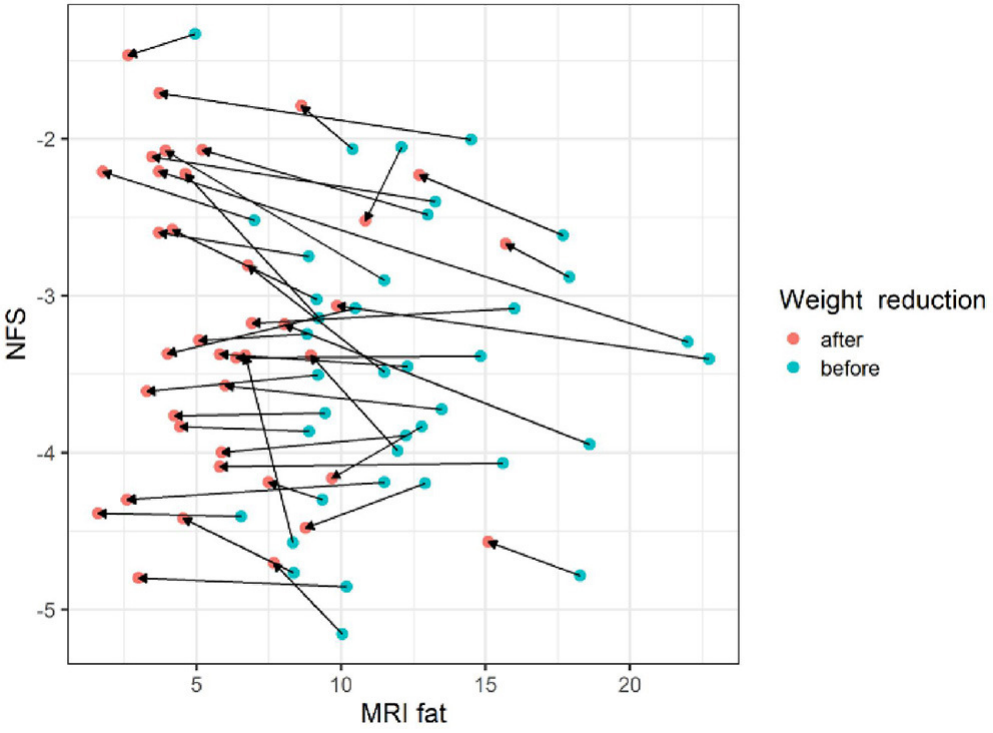
The changes of NFS according to MRI fat before and after body weight reduction. Regardless of the change in the MRS fat fraction according to the change in body weight, there was little change in the NFS value. MRS, magnetic resonance spectroscopy; NFS, NAFLD fibrosis score. Blue circle, before weight reduction; red circle and arrows, after weight reduction.

The gradient trend of the delta HSI/delta MRS graph was similar to that of the trend line, but there were some exceptional cases (Figure 3). No correlations were observed in the other two graphs (Figures 4, 5).

## Discussion

Previous studies have reported that MRS can accurately predict steatosis, although it remains controversial to what extent it can predict the degree of fibrosis (25, 31, 32). MRS is also non-invasive and more objective than biopsy assessments. In the present study, we investigated whether indices of hepatic steatosis and fibrosis can predict the extent of NAFLD after weight loss. Our findings indicated that there was no strong correlation between either of the two indices and the degree of steatosis, suggesting that the extent of change in hepatic steatosis due to weight reduction can only be roughly determined based on changes in HSI and FLI values.

Kahl et al. and Cuthbertson et al. reported the similar result that these HSI and FLI would be surrogate parameters for hepatic steatosis clinically, but could not replace MRS (33, 34).

Previous studies have suggested a correlation between HSI and fat fraction on ultrasonography; however, the R^2^ value was 0.334, and ultrasonography is a user-dependent method. Thus, the results were not clinically significant. Furthermore, the authors did not validate the correlation using liver histology findings (26). Although they attempted to determine the correlation between the index and MRS, no indices exhibited a quantitative correlation. Because we used the objective value for the fat fraction obtained via MRS as the reference value, our trends observed for HSI and FLI may be more reliable. Our regression analysis revealed a correlation for HSI only. Given that one can infer the extent of change on MRS based on the extent of change in HSI, this tool may allow clinicians to predict improvements in liver fat fraction without additional MRI or biopsy.

Liu et al. reported that FLI had a high diagnostic predictive rate for metabolic-associated fatty liver disease, but this may be due to triglyceride, gamma GT, which is highly correlated with metabolic diseases (35).

While NFS values increased, the change was not statistically significant, likely because we observed unexpected decreases in platelet levels, which are used in the calculation of NFS. Therefore, the results may have been clinically insignificant because the extent of change was not large. In addition, the NFS value was significantly smaller than the lower cutoff of −1.455 (−3.45 ± 0.89 before weight loss and −3.22 ± 0.94 after weight loss), and there was no donor with an index value exceeding the lower cutoff at either time point. Such findings indicate a degree of fibrosis without clinical significance. Since the study involved only those patients who had completed liver transplantation, our results were likely influenced by selection bias due to the inclusion of people with relatively healthy livers. For the same reason, most patients may have had low NFS values caused by simple steatosis. However, since this is also an index that suggests the probability of fibrosis, it is limited in that the true extent of fibrosis cannot be determined without a direct biopsy.

Our findings indicate that improvements in NAFLD can be predicted based only on routine laboratory examination results, which may aid in improving outcomes among donors with NAFLD. However, the arrow graph shows cases outside the normal gradient range. For clinical use, an additional understanding of the characteristics of exceptional cases will be required. In this study, we did not perform feature analysis of exceptional cases due to the small number of cases, although such analyses will be possible in larger-scale studies.

Our results also indicated that the fibrosis index was clinically and statistically insignificant; therefore, only steatosis could be identified. Pathological analyses should be performed before and after weight reduction to identify other factors that contribute to NAFLD, such as inflammation, ballooning injury, and fibrosis. The results of this study should also be validated based on pathological results.

Previous studies that have examined pathological findings at 1-year post-operatively have reported that recipient-related factors can influence the risk of recipient NAFLD after liver transplantation (36). To exclude these factors, future studies should examine pathology findings 7 days post-operatively, as this will help to ascertain the effect of weight reduction on liver engraftment in a linear fashion.

## Data Availability Statement

The raw data supporting the conclusions of this article will be made available by the authors, with permission of IRB.

## Ethics Statement

The studies involving human participants were reviewed and approved by Seoul National University Hospital. Written informed consent for participation was not required for this study in accordance with the national legislation and the institutional requirements.

## Author Contributions

JC: data collection, draft, and the final manuscript. YC: initial idea, study conceptualization, critical review, draft, and the final manuscript. SYH, SS, KH, and EH: data collection and draft review. JML: data collection, study conceptualization, critical review, draft, and review. SKH, NJY, KWL, and KSS: study conceptualization, critical review, and draft review. All authors contributed to the article and approved the submitted version.

## Conflict of Interest

The authors declare that the research was conducted in the absence of any commercial or financial relationships that could be construed as a potential conflict of interest.

## Publisher's Note

All claims expressed in this article are solely those of the authors and do not necessarily represent those of their affiliated organizations, or those of the publisher, the editors and the reviewers. Any product that may be evaluated in this article, or claim that may be made by its manufacturer, is not guaranteed or endorsed by the publisher.

## Abbreviations

NAFLD: non-alcoholic fatty liver disease
LDLT: living donor liver transplantation
MRS: magnetic resonance spectroscopy
ALT: alanine transaminase
AST: aspartate transaminase
GGT: gamma-glutamyl transpeptidase
FLI: fatty liver index
HSI: hepatic steatosis index
LAP: lipid accumulation product
ION: Index of Nash
NAFLD-LFS: NAFLD Liver Fat Score
APRI: AST to platelet ratio index
FIB-4: Fibrosis-4
ELF: Enhanced Liver Fibrosis
NFS: NAFLD Fibrosis Score
BMI: body mass index
TG: triglycerides
DM: diabetes mellitus.
